# Ratio of procalcitonin/Simpson’s dominance index predicted the short-term prognosis of patients with severe bacterial pneumonia

**DOI:** 10.3389/fcimb.2023.1175747

**Published:** 2023-07-03

**Authors:** Guoxian Sun, Weili Liu, Qingbin Zheng, Qing Shan, Hongling Hou

**Affiliations:** ^1^ Department of Infection Control, Affiliated Hospital of Yangzhou University, Yangzhou, China; ^2^ Department of Critical Care Unit, Affiliated Hospital of Yangzhou University, Yangzhou, China; ^3^ Department of Neurology, Affiliated Hospital of Yangzhou University, Yangzhou, China

**Keywords:** bacterial pneumonia, bronchoalveolar lavage fluid, procalcitonin, Simpson’s dominance index, predictive

## Abstract

**Objective:**

The aim of this study was to explore the predictive value of the ratio of procalcitonin (PCT) in serum to Simpson’s dominance index (SDI) in bronchoalveolar lavage fluid (BALF), in short-term prognosis of patients with severe bacterial pneumonia (SBP).

**Methods:**

This is a retrospective review of case materials of 110 patients with SBP who selected BALF metagenomic next-generation sequencing technique in the intensive care unit (ICU) of the Affiliated Hospital of Yangzhou University from January 2019 and July 2022. Based on the acute physiology and chronic health status score II, within 24 h after admission to the ICU, patients were divided into a non-critical group (*n* = 40) and a critical group (*n* = 70). Taking death caused by bacterial pneumonia as the endpoint event, the 28-day prognosis was recorded, and the patients were divided into a survival group (*n* = 76) and a death group (*n* = 34). The SDI, PCT, C-reactive protein (CRP), PCT/SDI, and CRP/SDI were compared and analyzed.

**Results:**

Compared with the non-critical group, the critical group had a higher PCT level, a greater PCT/SDI ratio, a longer ventilator-assisted ventilation time (VAVT), and more deaths in 28 days. Compared with the survivors, the death group had a higher PCT level, a lower SDI level, and a greater PCT/SDI ratio. The SDI level was significantly negatively correlated with the VAVT (*r* = −0.675, *p *< 0.05), while the PCT level, ratio of PCT/SDI, and ratio of CRP/SDI were remarkably positively correlated with VAVT (*r* = 0.669, 0.749, and 0.718, respectively, *p *< 0.05). The receiver operating characteristic (ROC) curves analysis showed that the area under ROC curves of PCT/SDI predicting patient death within 28 days was 0.851, followed by PCT + SDI, PCT, SDI, and CRP/SDI (0.845, 0.811, 0.778, and 0.720, respectively). The sensitivity and specificity of PCT/SDI for predicting death were 94.1% and 65.8%, respectively, at the optimal value (11.56). Cox regression analysis displayed that PCT/SDI (HR = 1.562; 95% CI: 1.271 to 1.920; *p* = 0.039) and PCT (HR = 1.148; 95% CI: 1.105 to 1.314; *p* = 0.015) were independent predictors of death in patients.

**Conclusion:**

The ratio of PCT/SDI was a more valuable marker in predicting the 28-day prognosis in patients with SBP.

## Introduction

Severe bacterial pneumonia (SBP) is one of the main causes of death in intensive care unit (ICU) patients, with a mortality rate of 15.5%–38.2% (Shi et al., 2019). Corresponding treatment guidelines recommend that early identification of the causative agent and treatment is an effective way to improve the patient’s prognosis (Kalil et al., 2016; Metlay et al., 2019). Patients with SBP have a severe inflammatory response in the lungs, with a massive release of inflammatory cytokines and a disruption of the original immune system balance, which leads to changes in the respiratory flora. Alterations in the respiratory flora, in turn, further promote disease progression and poor prognosis (Panzer et al., 2018). Thus, altered respiratory flora secondary to SBP may increase the risk of death in patients with pulmonary infections(Guo et al., 2021).

Understanding the composition of the respiratory flora is necessary to inform clinical decisions. Previous studies (Dima et al., 2019) have shown that respiratory flora is associated with the development and progression of several diseases, such as chronic obstructive pulmonary disease and acute respiratory distress syndrome. Simpson’s dominance index (SDI) is currently a sensitive indicator for studying differences in animal, plant, and soil flora, and has been shown to be a natural marker for bacterial infections in studies involving human respiratory flora (Langelier et al., 2018). It has been shown (Langelier et al., 2018) that SDI values are lower in patients with pulmonary infections compared to uninfected individuals. The more severe the patient’s lung bacterial infection, the lower the SDI value, as shown by the trend of SDI. The SDI decreases and PCT increases in SBP, and the ratio of the two indices (SDI/PCT) theoretically better reflects the predictive ability of the disease. Therefore, we proposed a method combining SDI and PCT for the first time exploratively.

The aim of the study was to analyze the ratio of serum PCT in ICU patients with bacterial pneumonia to SDI values obtained by the metagenomic next-generation sequencing technique (mNGS), and then compared with SDI, PCT, C-reactive protein (CRP), and CRP/SDI to evaluate the value of PCT/SDI in the short-term prognosis of ICU patients with SBP.

## Materials and methods

### Patients and study design

Adult patients (aged ≥ 18 years) with bacterial pneumonia selected for diagnosis with the aid of the bronchoalveolar lavage fluid (BALF) mNGS technique from the Affiliated Hospital of Yangzhou University between January 2019 and July 2022 were retrospectively reviewed. Age, gender, PCT, CRP, mNGS, patient outcomes and acute physiology, and chronic health evaluation (APACHE-II) within 24 h of admission were recorded. Diagnostic criteria for bacterial pneumonia refer to the guidelines for the diagnosis and treatment of hospital-acquired pneumonia and ventilator-associated pneumonia in adults in China (2018). Patients who met criterion 4 or any two or more of the first three criteria were included, as described below: (1) Newly developed cough, sputum, or aggravation of existing respiratory symptoms with purulent airway secretions, with or without chest pain; (2) body temperature >38°C; (3) peripheral blood leukocyte count (WBC) >10×10^9^/L or <4×10^9^/L, with or without left shift of nuclei; (4) lung imaging showing a new or progressive patchy infiltrative shadow, consolidation, or with or without pleural effusion. A total of 110 patients were enrolled in the study and bronchoscopy was performed to obtain BALF samples. The mNGS of BALF was used to detect bacteria. All patients were treated with appropriate antibiotics and steroid users were excluded. Based on APACHE-II within 24 h of admission to the ICU, all patients were divided into two groups: a non-critical group (APACHE-II <20, *n* = 40) and a critical group (APACHE-II ≥20, *n* = 70). The endpoints were bacterial pneumonia causing death of the patients, and 28-day prognosis was recorded. According to the endpoint outcomes, the patients were divided into two groups: a survival group (*n* = 76) and a death group (*n* = 34). This study was approved by the Ethics Committee of the Affiliated Hospital of Yangzhou University. Owing to a retrospective study, the ethics committee approved the waiver of informed consent. All research materials were conducted anonymously.

### Case definition and positive mNGS result criteria

Two independent physicians reviewed the anonymized data material for all patients. The likelihood of the infection source as suggested by clinical symptoms was determined. The bacteria that may have caused the infection were then identified based on the mNGS results. For rare reported bacteria, unless mNGS results were consistent with the clinical characteristics of the patient, the detected reads were classified as non-pathogenic bacteria sequences. This study excluded the case of viral, fungal, and atypical pathogenic bacteria infections. Finally, an infectious diagnosis was established.

Owing to the lack of interpretation criteria for mNGS, the following criteria were used to define positive bacterial results: (1) Pathogenic bacteria reported in the literature; (2) ≥3 reads of highly pathogenic bacteria at the species level; (3) >30% relative abundance at the genus level for opportunistic bacteria; and (4) ≥1 read for *Mycobacterium tuberculosis* and non-tuberculous mycobacteria.

### Sample processing

BALF was collected in accordance with standard operating procedures. The Vision Medicals’ Patho-NET technology was used to remove host gDNA. Then, we obtained up to 600-ml samples. DNA was extracted using a Pathogen DNA Kit (Tiangen Biotech, Beijing, China) following the manufacturer’s instructions, and DNA libraries were constructed by transposase-mediated ways (Vision Medicals, China). Prior to sequencing, the quality of the libraries was evaluated using the Qsep1 Biofragment Analyzer (BiOptic. Co., La Canada Flintridge, CA) to measure adapter and fragment size. The size of final library was 300 to 500 bp and the library concentration was greater than 0.5 ng/μl. Finally, the Nextseq 550 Dx sequencing platform (Illumina, San Diego, CA) was used for sequencing. High-quality data were obtained by removing short (less than 40 bp) reads. Human sequence was removed by mapping to human reference genome (hg38 and YH sequences) using Burrows–Wheeler Alignment. The remaining microbial sequence was classified by aligning to Microbial Genome Databases, which were downloaded from the NCBI Nucleotide and Genome databases. Finally, multiple parameters of bacteria, such as relative abundance, were exported, and the results were interpreted by microbiologists and clinicians.

### SDI calculation

The SDI was calculated using the number of bacterial nucleic acid sequences detected by mNGS with the following formula (Hunter, 1988):


$$\text{SDI}=1-\frac{\sum n\left(n-1\right)}{N\left(N-1\right)}$$

where *n* is the number of nucleic acid sequences detected for a strain and *N* is the total number of nucleic acid sequences detected for all strains.

### Statistical analysis

Mann–Whitney *U* test (non-normally distributed variables) and independent-samples *t*-tests (normally distributed variables) were utilized to compare the quantitative data between the groups. Chi-square tests were used to compare categorical variables. The Spearman method was utilized for correlation analysis. Binary logistic regression analysis was applied to identify independent risk factors associated with patient death in 28 days. Receiver operating curves (ROCs) were used to obtain cutoff values for the best sensitivity and specificity of factors for patient death in 28 days. Multivariate Cox regression analysis was carried out to explore whether the variables can effectively predict the short-term prognosis of patients. All analyses were performed using SPSS 17.0 (SPSS Inc., Chicago, IL, USA), and figures were constructed using MedCalc version 20 software (MedCalc Software Ltd., Ostend, Belgium). *p*< 0.05 was considered significant.

## Results

### Patient characteristics

A total of 110 patients with SBP (70 male and 40 female patients) with a median age of 65 (62, 71) years were enrolled in this study. The median SDI, PCT, and CRP for all patients were 0.460 (0.350, 0.588), 5.050 (3.845, 7.813) ng/ml, and 21.320 (16.863, 27.533) mg/L, respectively, and the median duration of ventilator-assisted ventilation time (VAVT) was 9.0 (7.0, 14.0) days. All patients were followed up with a 28-day prognosis and 34 patients eventually died. The bacterial test results and clinical characteristics of patients are shown in **Table 1**.

**Table 1:** Clinical characteristics and laboratory and bacterial results of patients.

Characteristics	Overall (*n* = 110)
Gender (male/female)	70/40
Age, years	65.0 (62.0, 71.0)
VAVT, days	9.0 (7.0, 14.0)
Laboratory examination	
SDI	0.460 (0.350, 0.588)
PCT, ng/ml	5.050 (3.845, 7.813)
CRP, mg/L	21.320 (16.863, 27.533)
Bacteria	
* Acinetobacter baumannii*	20
* Klebsiella pneumoniae*	16
* Pseudomonas aeruginosa*	14
* Streptococcus pneumoniae*	13
* Escherichia coli*	11
* Burkholderia cepacia*	9
* Stenotrophomonas maltophilia*	7
* Aspergillus*	5
* Staphylococcus aureus*	5
* Listeria*	5
* Haemophilus influenzae*	4
* Mycoplasma*	4
* Pneumocystis carinii*	3
* Aerobacter cloacae*	1
* Mycobacterium tuberculosis*	1
* Legionella pneumophila*	1

Continuous variables are presented as median and interquartile range; binary variables are presented as number and percentage. VAVT, ventilator-assisted ventilation time; SDI, Simpson’s dominance index; PCT, procalcitonin; CRP, C-reactive protein.

### Comparison of the indicators between the critical group and the non-critical group

The critical patients had significantly lower SDI levels; higher PCT/SDI, PCT, and CRP/SDI values; longer VAVT; and more deaths compared with the non-survival group (*p *< 0.01), while the age, gender, and CRP were not statistically significant (*p >* 0.05) (**Table 2**).

**Table 2:** Various indicators of the critical group and non-critical group.

Indicators	Non-critical group (*n* = 40)	Critical group (*n* = 70)	*p*-value
Gender (male/female)	28/12	42/28	0.294
Age, years	67.5 (62.0, 72.0)	64.0 (62.0, 70.0)	0.076
SDI	0.625 (0.553, 0.663)	0.410 (0.270, 0.460)	<0.001^*^
PCT/SDI	6.090 (5.113, 8.283)	17.010 (11.640, 24.690)	<0.001^*^
PCT, ng/ml	3.775 (3.388, 4.690)	7.120 (4.925, 8.240)	<0.001^*^
CRP/SDI	33.360 (28.900, 42.520)	65.810 (45.753, 97.958)	<0.001^*^
CRP, mg/L	24.165 (22.245, 26.725)	24.170 (21.433, 30.563)(20.125,28.220)	0.177
VAVT, days	7.0 (4.8, 8.3)	11.0 (8.0, 16.8)	<0.001^*^
28-day prognosis(die/survival)	6/34	28/42	0.006^*^

Continuous variables are presented as median and interquartile range; binary variables are presented as number and percentage. VAVT, ventilator-assisted ventilation time; SDI, Simpson’s dominance index; PCT, procalcitonin; CRP, C-reactive protein.

### Comparison of the indicators between the death group and the survival group

Patients with death had higher PCT/SDI, PCT, and CRP/SDI values, and lower SDI values compared with the survival group (all *p *< 0.05), while the age, gender, and CRP were not statistically significant (*p >* 0.05) (**Table 3**).

**Table 3:** Various indicators of the survivor group and death group.

Indicators	Survivor group (*n* = 76)	Death group (*n* = 34)	*p*-value
Gender (male/female)	52/24	18/16	0.119
Age, years	65.5 (61.0, 71.0)	64.5 (63.0, 69.3)	0.314
SDI	0.485 (0.430, 0.630)	0.320 (0.270, 0.460)	<0.001^*^
PCT/SDI	9.250 (5.800, 16.500)	21.420 (13.798, 33.900)	<0.001^*^
PCT, ng/ml	4.700 (3.640, 5.970)	7.900 (5.403, 9.970)	<0.001^*^
CRP/SDI	44.140 (23.860, 63.070)	76.440 (42.560, 118.008)	0.004^*^
CRP, mg/L	21.165 (15.030, 27.120)	22.020 (20.120, 29.230)	0.064

Continuous variables are presented as median and interquartile range; binary variables are presented as number and percentage. SDI, Simpson’s dominance index; PCT, procalcitonin; CRP, C-reactive protein.

### Correlation of indicators with the VAVT

**Figure 1:**
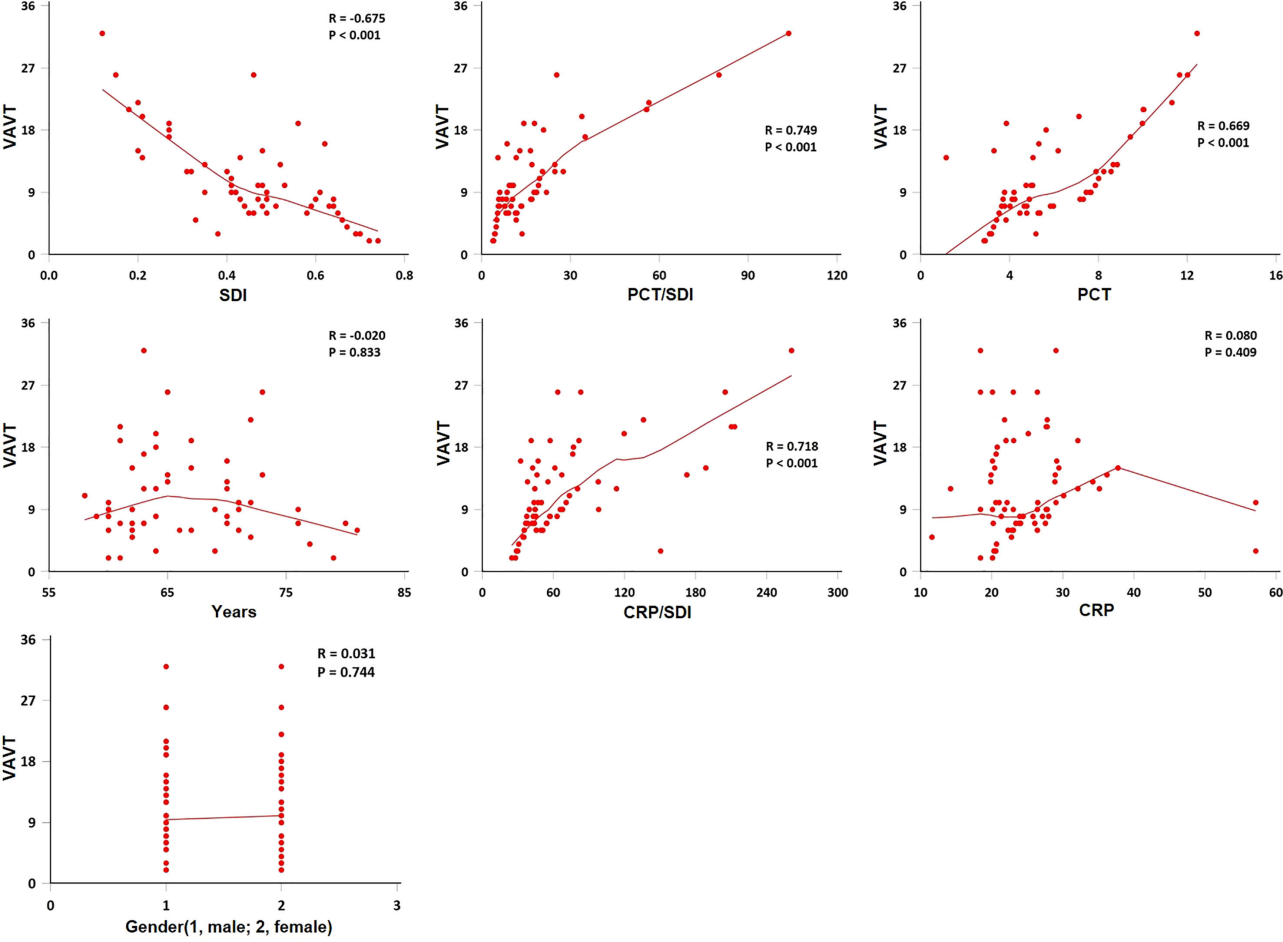
Spearman method for correlation of ventilator-assisted ventilation time (VAVT) with indexes. The VAVT by age, gender, Simpson’s diversity index (SDI), procalcitonin (PCT), C-reactive protein (CRP), PCT/SDI, and CRP/SDI (*n* = 110). SDI was negatively correlated with VAVT (*p *< 0.001), and PCT, PCT/SDI, and CRP/SDI were positively correlated with VAVT (*p *< 0.001). Gender, age and CRP were not correlated with VAVT (*p* > 0.05).

SDI was negatively correlated with VAVT (Spearman’s correlation coefficient; *r* = −0.675, *p *< 0.001), and PCT, PCT/SDI, and CRP/SDI were positively correlated with VAVT (Spearman’s correlation coefficient; *r* = 0.669, 0.749, and 0.718, respectively, *p *< 0.001). Gender, age, and CRP were not correlated with VAVT (Spearman’s correlation coefficient; *r* = 0.031, −0.020, and 0.080, respectively, *p >* 0.05) (**Figure 1**).

### ROC curve analysis for indicators

**Figure 2:**
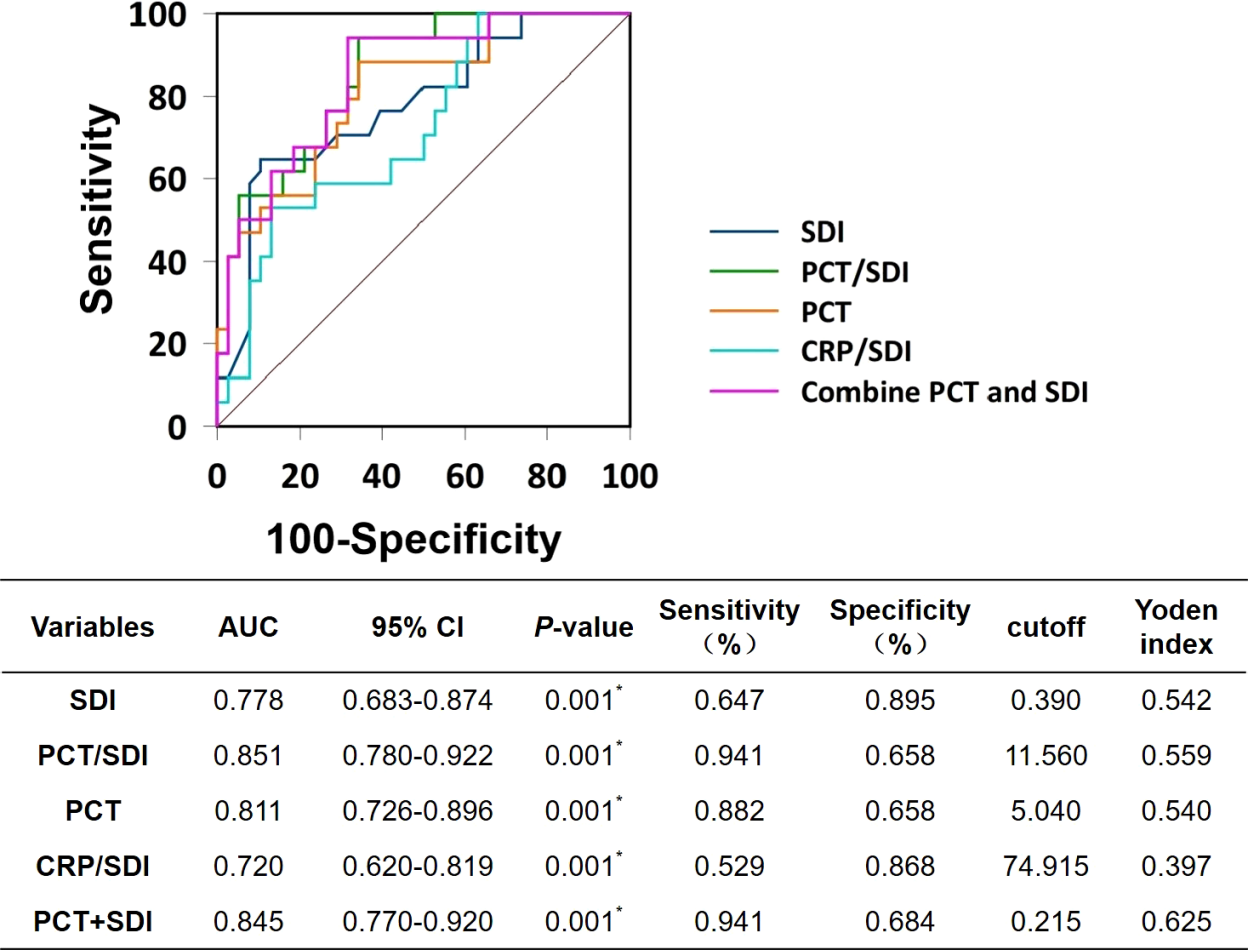
Receiver operating characteristic curves. SDI, Simpson’s dominance index; PCT, procalcitonin; CRP, C-reactive protein; AUC, area under the ROC curves.

We included statistically significant PCT, SDI, PCT/SDI, PCT+SDI, and CRP/SDI in the survival group compared with the death group in the ROC curve analysis. The results showed that the area under the ROC curves for PCT/SDI predicting patient death in 28 days was 0.851, followed by PCT+SDI (0.845), PCT (0.811), SDI (0.778), and CRP/SDI (0.720). Pairwise comparisons of the area under the ROC curve for each variables showed that SDI ~ PCT/SDI, *Z* = 1.191, *p* = 0.234; SDI ~ PCT, *Z* = 0.810, *p* = 0.418; SDI ~ CRP/SDI, *Z* = 1.910, *p* = 0.056; SDI ~ PCT+SDI, *Z* = 1.908, *p* = 0.056; PCT/SDI ~ PCT, *Z* = 2.081, *p* = 0.038; PCT/SDI ~ CRP/SDI, *Z* = 0.603, *p* = 0.547; PCT ~ CRP/SDI, *Z* = 2.058, *p* = 0.040; PCT ~ PCT+SDI, *Z* = 0.974, *p* = 0.330; CRP/SDI ~ PCT/SDI, *Z* = 3.531, *p *< 0.001. When the cutoff value of PCT/SDI was 11.56, the best sensitivity for predicting patient death in 28 days was 94.1% and the specificity was 65.8% (**Figure 2**).

### Multivariate Cox regression analysis for 28-day survival in patients with SBP

**Figure 3:**
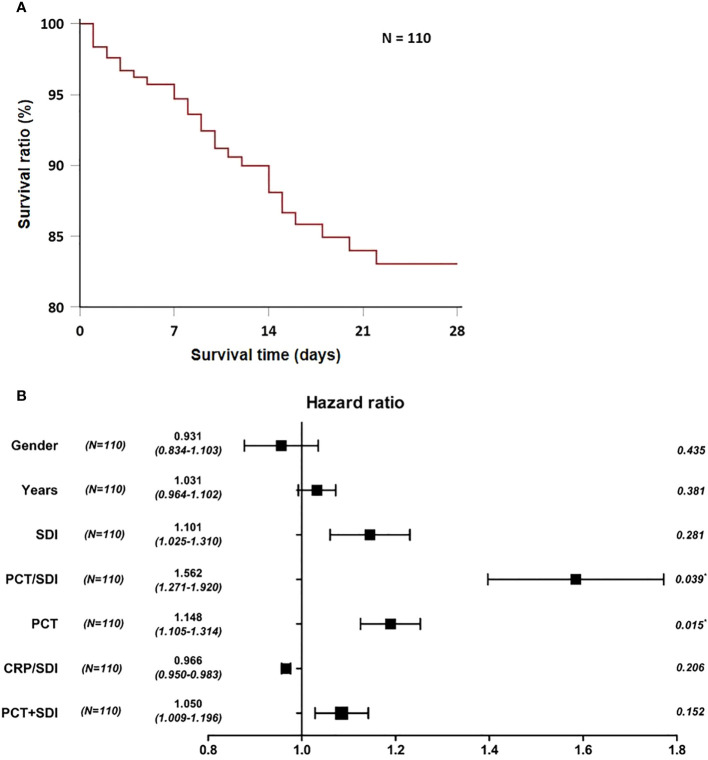
Construction of short-term prognostic features of patients with bacterial pneumonia. **(A)** Twenty-eight-day survival curve for patients with SBP. **(B)** Forest plots of multivariate Cox regression analysis for 28-day mortality in patients with SBP. SDI, Simpson’s dominance index; PCT, procalcitonin; CRP, C-reactive protein.

The survival time is defined as the time from the patient’s admission to the ICU to death or the end of follow-up. After removing irrelevant features, age, sex, SDI, PCT/SDI, PCT, CRP/SDI, and PCT+SDI were taken as independent variables, and multivariate Cox regression analysis was performed. The multivariate Cox regression analysis showed PCT/SDI and PCT as continuous variables and were independent risk factors for 28-day death in patients with SBP (**Figures 3A, B**).

## Discussion

It is now generally accepted that the human body has a complex micro-ecosystem in all the cavities that are connected to the outside world. The imbalance of the microecosystem in the luminal tract and the inflammatory state due to bacterial infection are the main factors for the poor prognosis of patients with severe infections. Multiple studies on the relationship between intestinal flora and disease have shown that the incidence of acute respiratory distress syndrome and ventilator-associated pneumonia is significantly higher once intestinal flora is imbalanced (Dickson et al., 2020; Zanza et al., 2022). In many cases, the pathological changes of the lung, such as parenchymal fibrosis, is considered to be associated with altered microbial communities in the patient’s lungs (Dickson and Huffnagle, 2015; Woo et al., 2020). The composition of the intrapulmonary microbial community varies significantly with the progression of the disease. ICU patients with SBP often have advanced age, underlying diseases, and immune deficiencies that can lead to physiological dysfunction and disruption of the body’s microecological balance. Previous studies have shown that alterations in microbial communities increase the incidence of infectious diseases through their metabolite-mediated immune regulation (Segal et al., 2017). Further studies suggest that an imbalanced microbial community increases immune-related metabolite production pathways, such as the pentose phosphate pathway and the glycolytic pathway (Hong et al., 2021). As can be expected, alterations in the intrapulmonary microbial community play a special role in the recognition and treatment of infectious diseases. Therefore, simultaneous monitoring of intrapulmonary microbial communities and inflammatory indicators in patients with SBP may be of more clinical value.

SDI was proposed by the British scholar Simpson in 1949 as a measure of the number of biological species within a species community and the relative abundance among species, the principle of which can be derived from probability theory (Simpson, 1949). SDI can reflect the status and role of dominant species in the community, and the larger its value, the higher the ecological dominance. According to previous research (Yang et al., 2018; Pimple, 2020), SDI can elucidate differences in species (flora) such as animals and plants within a given region; however, most recent studies have dealt with human respiratory and intestinal flora (Langelier et al., 2018). Clinical studies have shown (Pimple, 2020) that a subgroup comparison of 22 adult hospitalized patients after hematopoietic stem cell transplantation had significantly lower SDI values in the infected group relative to the uninfected group (*p* = 0.017). This is similar to the results of our previous unpublished study with a small sample. PCT and CRP are commonly used biomarkers that have the potential to differentiate infectious and non-infectious inflammatory conditions. For the severity and prognosis of bacterial pneumonia, in conjunction with other clinical examination, PCT levels have been shown to be more advantages than CRP (Covington et al., 2018). The results of this study showed that the PCT values of patients in the survival and non-critical groups were significantly lower than those in the death and critical groups (*p*< 0.001), while CRP was not significantly different in any of the groups (*p >* 0.05). The PCT levels of patients with bacterial pneumonia were positively correlated with the VAVT. The greater the PCT value, the more severe the disease and the higher the risk of death.

Previous research have shown that the ratio of PCT and CRP to indicators such as serum prealbumin or erythrocyte sedimentation rate (ESR) has clinical significance in the diagnosis, management, and prognosis, such as the study by Christopher et al. (2021), which showed that ESR/CRP helps to determine the duration of periprosthetic joint infection and informs the physician’s treatment choice. Another study showed that serum prealbumin/PCT was negatively associated with time in intensive care and death, and could be used as an indicator of patient severity and short-term prognosis (Wang et al., 2022). Given the high and low variability of PCT, CRP, and SDI in the context of bacterial infection in patients, the ratio of inflammatory indexes to SDI was used exploratively in this study. In contrast to PCT for short-term prognosis of patients with bacterial pneumonia, SDI values were taken independent of a variety of clinical factors such as renal insufficiency, autoimmune disease, and neutropenia (Covington et al., 2018). In addition, early and effective antimicrobial therapy in patients can lead to a decrease in the accuracy of PCT as an indicator of prognosis. Therefore, PCT/SDI can be more sensitive to the trend of ratio results. According to our study data, PCT/SDI and CRP/SDI were higher in the critical group than in the non-critical group, and the former group had longer VAVT and more cases of death within 28 days. The SDI values of patients in the death group were lower than those in the survival group, while PCT/SDI and CRP/SDI were higher than those in the survival group, and PCT/SDI was positively correlated with the VAVT. SDI was negatively correlated with the VAVT; SDI was negatively correlated with the VAVT, while age, sex, and CRP were not statistically significant when compared between groups, suggesting that PCT/SDI and CRP/SDI were correlated with the severity of disease in patients with SBP in the ICU.

The ROC curve showed that the area under the curve of PCT/SDI for predicting 28-day death in patients with SBP was 0.851, which was better than PCT (0.811), SDI (0.778), CRP/SDI (0.720), and PCT+SDI (0.845), indicating that the highest accuracy in determining the 28-day prognosis of patients with SBP was observed with a PCT/SDI ratio of 11.56, with a sensitivity and specificity of 94.1% and 65.8%, respectively. The multivariate Cox regression analysis demonstrated that PCT/SDI (HR = 1.562; 95% CI: 1.271 to 1.920; *p* = 0.039) and PCT (HR = 1.148; 95% CI: 1.105 to 1.134; *p* = 0.015) were independent risk factors for the 28-day death of patients with bacterial pneumonia in the ICU. Given the impact of multiple diseases on PCT values, such as renal insufficiency, organ transplantation, and autoimmune diseases, it is reasonable to confirm that PCT/SDI can be a more sensitive indicator of a patient’s short-term prognosis (Covington et al., 2018).

Some limitations of the study should be considered. First, given its retrospective nature, only 110 patients was included. Second, patients were not divided into subgroups according to their ventilation mode, such as invasive ventilation and noninvasive ventilation. These factors may affect the validity of the PCT/SDI ratio in determining the prognosis of patients with SBP. Third, considering the study methodology and the cost of SDI, this study did not perform dynamic monitoring and evaluation of PCT/SDI to further explore the dynamic change pattern of the index. We expect the cost of SDI to decrease, so that the sample size can be expanded to further evaluate PCT/SDI.

## Conclusions

In conclusion, although the PCT/SDI ratio is a tool to predict the 28-day prognosis of patients with SBP and may help to reduce the impact of various diseases on the monitoring results, the ratio is not a perfect method to replace PCT for clinical use. However, as a monitoring method with a sensitivity close to 90%, this method will still help clinicians to assess the short-term prognosis of patients with SBP. Given that the accuracy of PCT and CRP are affected by several factors, the use of antimicrobial drugs can also lead to a decrease in the accuracy of PCT and CRP results. This study indicates the high potential value of PCT/SDI ratio as a method to assess the short-term prognosis of these patients.

## Data availability statement

The datasets presented in this study can be found in online repositories. The names of the repository/repositories and accession number(s) can be found below CNSA, CNP0003255.

## Ethics statement

The studies involving human participants were reviewed and approved by Ethics Committee of Affiliated Hospital of Yangzhou University. Written informed consent for participation was not required for this study in accordance with the national legislation and the institutional requirements.

## Author contributions

HH and QS conceived and designed this study. WL, QZ, and GS collected the data. GS drafted and critically revised the manuscript. All authors have read and approved the final manuscript. All authors contributed to the article and approved the submitted version.
