# Supplementary material for: Ratio of procalcitonin/Simpson’s dominance index predicted the short-term prognosis of patients with severe bacterial pneumonia
Source: Front Cell Infect Microbiol. 2023 Jul 3;13:1175747. doi: 10.3389/fcimb.2023.1175747 (PMC10350521; doi:10.3389/fcimb.2023.1175747)
Supplement: Supplementary file 1 [file Table_1.docx]

**Supplementary Table 1** The relative abundance of the different pathogenic bacteria.

| Patient ID | NGS result | Relative abundance  (%) | Patient ID | NGS result | Relative abundance  (%) |
| --- | --- | --- | --- | --- | --- |
| 1 | *Acinetobacter baumannii* | 30.3 | 56 | *Acinetobacter baumannii* | 29.8 |
| 2 | *Klebsiella pneumoniae* | 19.5 | 57 | *Acinetobacter baumannii* | 42.1 |
| 3 | *Pseudomonas aeruginosa* | 36.7 | 58 | *Mycoplasma* | 95.8 |
| 4 | *Streptococcus pneumoniae* | 79.3 | 59 | *Mycoplasma* | 99.1 |
| 5 | *Escherichia coli* | 60.1 | 60 | *Pseudomonas aeruginosa* | 70.3 |
| 6 | *Streptococcus pneumoniae* | 30.2 | 61 | *Acinetobacter baumannii* | 37.4 |
| 7 | *Acinetobacter baumannii* | 36.2 | 62 | *Klebsiella pneumoniae* | 39.2 |
| 8 | *Acinetobacter baumannii* | 46.4 | 63 | *Klebsiella pneumoniae* | 40.2 |
| 9 | *Burkholderia cepacia* | 29.3 | 64 | *Pseudomonas aeruginosa* | 30.8 |
| 10 | *Stenotrophomonas maltophilia* | 40.2 | 65 | *Acinetobacter baumannii* | 43.2 |
| 11 | *Aspergillus* | 98.7 | 66 | *Streptococcus pneumoniae* | 37.3 |
| 12 | *Mycoplasma* | 98.9 | 67 | *Pseudomonas aeruginosa, Stenotrophomonas maltophilia* | 29.2, 37.4 |
| 13 | *Staphylococcus aureus, Burkholderia cepacia* | 29.2, 40.5 | 68 | *Pseudomonas aeruginosa, Stenotrophomonas maltophilia* | 17.7, 39.3 |
| 14 | *Listeria* | 99.0 | 69 | *Acinetobacter baumannii* | 38.7 |
| 15 | *Haemophilus influenzae* | 31.1 | 70 | *Pneumocystis carinii* | 99.0 |
| 16 | *Streptococcus pneumoniae* |  | 71 | *Pneumocystis carinii* | 98.6 |
| 17 | *Streptococcus pneumoniae* |  | 72 | *Acinetobacter baumannii* | 33.2 |
| 18 | *Streptococcus pneumoniae* | 50.1 | 73 | *Pneumocystis carinii* | 99.3 |
| 19 | *Acinetobacter baumannii* | 34.9 | 74 | *Klebsiella pneumoniae* | 29.1 |
| 20 | *Escherichia coli* | 36.3 | 75 | *Legionella pneumophila* | 99.3 |
| 21 | *Klebsiella pneumoniae, Stenotrophomonas maltophilia* | 29.3, 40.1 | 76 | *Acinetobacter baumannii* | 35.7 |
| 22 | *Acinetobacter baumannii* | 41.1 | 77 | *Mycobacterium tuberculosis* | 97.3 |
| 23 | *Acinetobacter baumannii* | 22.4 | 78 | *Pseudomonas aeruginosa* | 31.0 |
| 24 | *Acinetobacter baumannii* | 60.1 | 79 | *Acinetobacter baumannii* | 29.8 |
| 25 | *Escherichia coli* | 37.2 | 80 | *Klebsiella pneumoniae, Stenotrophomonas maltophilia* | 33.2, 47.0 |
| 26 | *Klebsiella pneumoniae* | 19.7 | 81 | *Pseudomonas aeruginosa, Haemophilus influenzae* | 29.3, 38.4 |
| 27 | *Escherichia coli* | 42.3 | 82 | *Acinetobacter baumannii* | 36.1 |
| 28 | *Klebsiella pneumoniae, Stenotrophomonas maltophilia* | 70.2, 28.3 | 83 | *Escherichia coli* | 30.1 |
| 29 | *Burkholderia cepacia* | 33 | 84 | *Klebsiella pneumoniae* | 39.3 |
| 30 | *Burkholderia cepacia, Streptococcus pneumoniae* | 40.6, 31.3 | 85 | *Klebsiella pneumoniae* | 35.9 |
| 31 | *Burkholderia cepacia* | 61.4 | 86 | *Listeria* | 99.2 |
| 32 | *Escherichia coli* | 42.3 | 87 | *Pseudomonas aeruginosa* | 70.7 |
| 33 | *Escherichia coli* | 38 | 88 | *Escherichia coli* | 39.1 |
| 34 | *Klebsiella pneumoniae* | 56.3 | 89 | *Escherichia coli* | 30.8 |
| 35 | *Escherichia coli* | 28.4 | 90 | *Burkholderia cepacia* | 39.0 |
| 36 | *Escherichia coli* | 49.2 | 91 | *Listeria* | 99.2 |
| 37 | *Acinetobacter baumannii, Streptococcus pneumoniae* | 36.2, 19.9 | 92 | *Burkholderia cepacia* | 31.1 |
| 38 | *Stenotrophomonas maltophilia* | 37.1 | 93 | *Acinetobacter baumannii* | 33.2 |
| 39 | *Aspergillus* | 99.3 | 94 | *Burkholderia cepacia* | 39.0 |
| 40 | *Klebsiella pneumoniae* | 39.3 | 95 | *Staphylococcus aureus* | 18.7 |
| 41 | *Klebsiella pneumoniae* | 62.6 | 96 | *Aspergillus* | 99.1 |
| 42 | *Pseudomonas aeruginosa* | 56.7 | 97 | *Acinetobacter baumannii* | 60.4 |
| 43 | *Staphylococcus aureus* | 28.7 | 98 | *Aerobacter cloacae* | 30.2 |
| 44 | *Klebsiella pneumoniae* | 18.5 | 99 | *Streptococcus pneumoniae* | 36.0 |
| 45 | *Listeria* | 98.4 | 100 | *Staphylococcus aureus* | 16.7 |
| 46 | *Pseudomonas aeruginosa* | 57.1 | 101 | *Streptococcus pneumoniae* | 30.3 |
| 47 | *Staphylococcus aureus* | 15.7 | 102 | *Burkholderia cepacia* | 51.1 |
| 48 | *Acinetobacter baumannii* | 29.6 | 103 | *Pseudomonas aeruginosa* | 37.4 |
| 49 | *Listeria* | 98.1 | 104 | *Streptococcus pneumoniae* | 29.0 |
| 50 | *Haemophilus influenzae* | 27.4 | 105 | *Aspergillus* | 96.3 |
| 51 | *Pseudomonas aeruginosa* | 52.7 | 106 | *Streptococcus pneumoniae* | 29.1 |
| 52 | *Klebsiella pneumoniae* | 39.2 | 107 | *Aspergillus* | 97.3 |
| 53 | *Mycoplasma* | 97.2 | 108 | *Pseudomonas aeruginosa* | 49.3 |
| 54 | *Klebsiella pneumoniae* | 39.0 | 109 | *Pseudomonas aeruginosa* | 62.0 |
| 55 | *Haemophilus influenzae* | 47.2 | 110 | *Streptococcus pneumoniae* | 41.3 |

**Supplementary Table 2** The relative abundance of the different pathogenic bacteria between critical and non-critical groups.

| Bacteria | Non-critical group (n=40) | | |  | Critical group (n=70) | | |
| --- | --- | --- | --- | --- | --- | --- | --- |
|  | N |  | relative abundance  (%) |  | N |  | relative abundance  (%) |
| *Acinetobacter baumannii* | 6 |  | 34.5 (33.2, 36.0) |  | 14 |  | 36.8 (29.9, 40.9) |
| *Klebsiella pneumoniae* | 4 |  | 34.6 (32.2, 36.8) |  | 12 |  | 39.2 (26.9, 44.2) |
| *Pseudomonas aeruginosa* | 6 |  | 43.4 (32.6, 58.8) |  | 8 |  | 44.7 (30.4, 56.8) |
| *Streptococcus pneumoniae* | 5 |  | 35.3 (29.1, 46.0) |  | 8 |  | 36.8 (31.0, 47.3) |
| *Escherichia coli* | 3 |  | 30.1, 39.1, 30.8 |  | 8 |  | 40.2 (37.0, 44.0) |
| *Burkholderia cepacia* | 4 |  | 39.0 (37.0, 42.0) |  | 5 |  | 40.6 (40.5, 42.9) |
| *Stenotrophomonas maltophilia* | 1 |  | 47.0 |  | 6 |  | 38.4 (37.2, 39.9) |
| *Aspergillus* | 3 |  | 99.1, 96.3, 97.3 |  | 2 |  | 98.7, 99.3 |
| *Staphylococcus aureus* | 2 |  | 18.7, 16.7 |  | 3 |  | 28.7, 15.7, 29.2 |
| *Listeria* | 2 |  | 99.2, 99.4 |  | 3 |  | 99.0, 98.4, 98.1 |
| *Haemophilus influenzae* | 1 |  | 38.4 |  | 3 |  | 31.1, 27.4, 47.2 |
| *Mycoplasma* | 0 |  | — |  | 4 |  | 98.1 (96.9, 99.0) |
| *Pneumocystis carinii* | 3 |  | 99.0, 99.3, 98.6 |  | 0 |  | — |
| *Aerobacter cloacae* | 1 |  | 30.2 |  | 0 |  | — |
| *Mycobacterium tuberculosis* | 1 |  | 97.3 |  | 0 |  | — |
| *Legionella pneumophila* | 1 |  | 99.3 |  | 0 |  | — |

N: number of patients. Relative abundance is presented as [M (P25, P75)]. Relative abundances are all listed when the number of patients is less than 4.

The names of the repository/repositories and accession number(s) can be found below: CNSA, CNP0003255.
